# Changes in the Carbon Metabolism of *Escherichia coli* During the Evolution of Doxycycline Resistance

**DOI:** 10.3389/fmicb.2019.02506

**Published:** 2019-11-01

**Authors:** Yiwen Yang, Jiandui Mi, Jiadi Liang, Xindi Liao, Baohua Ma, Yongde Zou, Yan Wang, Juanboo Liang, Yinbao Wu

**Affiliations:** ^1^College of Animal Science, National Engineering Research Center for Breeding Swine Industry, South China Agricultural University, Guangzhou, China; ^2^Ministry of Agriculture Key Laboratory of Tropical Agricultural Environment, South China Agricultural University, Guangzhou, China; ^3^Key Laboratory of Chicken Genetics, Breeding and Reproduction, Ministry of Agriculture, Guangzhou, China; ^4^Guangdong Provincial Key Laboratory of Agro-Animal Genomics and Molecular Breeding, South China Agriculture University, Guangzhou, China; ^5^Nanhai Office of Foshan Customs House, Foshan, China; ^6^Laboratory of Animal Production, Institute of Tropical Agriculture, Universiti Putra Malaysia, Serdang, Malaysia

**Keywords:** carbon metabolism, evolution, antibiotic resistance, DOX, *Escherichia coli*

## Abstract

Despite our continuous improvement in understanding the evolution of antibiotic resistance, the changes in the carbon metabolism during the evolution of antibiotic resistance remains unclear. To investigate the evolution of antibiotic resistance and the changes in carbon metabolism under antibiotic pressure, *Escherichia coli* K-12 was evolved for 38 passages under a concentration gradient of doxycycline (DOX). The 0th-passage sensitive strain W0, the 20th-passage moderately resistant strain M20, and the 38th-passage highly resistant strain E38 were selected for the determination of biofilm formation, colony area, and carbon metabolism levels, as well as genome and transcriptome sequencing. The MIC of DOX with *E. coli* significantly increased from 4 to 96 μg/ml, and the IC_50_ increased from 2.18 ± 0.08 to 64.79 ± 0.75 μg/ml after 38 passages of domestication. Compared with the sensitive strain W0, the biofilm formation amount of the resistant strains M20 and E38 was significantly increased (*p* < 0.05). Single-nucleotide polymorphisms (SNPs) were distributed in antibiotic resistance-related genes such as ribosome targets, cell membranes, and multiple efflux pumps. In addition, there were no mutated genes related to carbon metabolism. However, the genes involved in the biosynthesis of secondary metabolites and carbon metabolism pathway were downregulated, showing a significant decrease in the metabolic intensity of 23 carbon sources (*p* < 0.05). The results presented here show that there may be a correlation between the evolution of *E. coli* DOX resistance and the decrease of carbon metabolism, and the mechanism was worthy of further research, providing a theoretical basis for the prevention and control of microbial resistance.

## Introduction

Antibiotics are one of the great discoveries of contemporary humans. They play an important role in the treatment of various infectious diseases and have saved countless lives. At the same time, antibiotics also have the function of promoting animal production (Angelakis et al., 2013; Le Roy et al., 2019). Therefore, antibiotics are widely used in clinics and livestock production. However, the widespread use of antibiotics has also brought about microbial resistance. The use of antibiotics can cause stress on microorganisms and promote the evolution of microbial resistance (Xiong et al., 2015; Sui et al., 2019), leading to the risk of environmentally resistant bacteria (Schwartz et al., 2003). When *E. coli* was inoculated into media containing a trimethoprim gradient, the resistance of *E. coli* to trimethoprim increased gradually, and the minimum inhibitory concentration (MIC) of trimethoprim increased 1,000-fold on the 12th day (Baym et al., 2016). In addition, when *E. coli* MGY, EPEC, and KLY were cultured for 11–14 passages in 50 μg/ml ampicillin-containing medium, the MIC of ampicillin in these strains increased 7-fold (Levin-Reisman et al., 2017).

Under the pressure of antibiotic selection, there are two ways to evolve microbial resistance, gene mutation, and gene level transfer (Hua et al., 2017; Lukacisinova and Bollenbach, 2017). Among them, the gene mutation is the most important way. For example, single mutations in *E. coli* at the Gly81Asp, Asp82Gly, and Ser83Leu loci of *gyrA* gene can produce resistance to ciprofloxacin (Truong et al., 1997). However, this mutation may also cause changes in other functions of the bacteria (Dahlberg and Chao, 2003; Melnyk et al., 2015). Trimethoprim can induce mutations in the folic acid synthesis-related genes of *E. coli*, thereby affecting folic acid metabolism (Toprak et al., 2011). Ciprofloxacin induces mutations in *staphylococcal* genes, leading to the resistance evolution and a decline in growth performance (Dengler Haunreiter et al., 2019). Folate metabolism is a carbon metabolism, and carbon metabolism directly affects the growth characteristics of bacteria (Cristiano-Fajardo et al., 2019). Therefore, carbon metabolism, the important metabolic pathway of microbes, may be related to the evolution of antibiotic resistance. However, the relationship between metabolic constraints and antibiotic resistance evolution is poorly understood.

Tetracycline antibiotics not only exhibit favorable therapeutic effects against animal infections, but also play an active role in promoting animal growth and ensuring the healthy development of animals that are widely used in husbandry. According to statistical analysis, tetracycline antibiotic used in China each year accounts for 7% of total antibiotic use (Zhang et al., 2015). Tetracycline antibiotics, which are used in large quantities, remain in the environment and affect environmental microorganisms (Yan et al., 2018; Keijser et al., 2019). However, there have been no systematic studies on the evolution of resistance to tetracycline antibiotics. DOX, a synthetic tetracycline, is more potent than the natural tetracycline and is one of the most commonly used tetracycline antibiotics. Therefore, DOX was selected as the research subject in this paper to systematically study the evolution of resistance.

First, the trend of *E. coli* resistance under gradient DOX stress was studied. *E. coli* K-12 was evolved *via* growth in different concentrations of DOX for 38 successive subcultures, and different strains of domesticated passages were obtained to detect resistance to DOX. Then, the 0th-passage sensitive strain W0, the 20th-passage moderately resistant strain M20, and the 38th-passage highly resistant strain E38 were selected for determination of biofilm formation, colony area, and carbon metabolism levels, as well as genome and transcriptome sequencing. The purposes of this study were to identify the changes in the carbon metabolism of *E. coli* during the evolution of DOX resistance.

## Materials and Methods

### Bacterial Strains and Culture Conditions

*E. coli* K-12 BW25113, purchased from the China Center of Industrial Culture Collection (CICC), was used as the wild-type (WT) strain throughout this study. The growth medium was a standard Luria-Bertani (LB) broth medium with 10.0 g/L peptone, 5.0 g/L sodium chloride, 1.0 g/L dextrose, and 5.0 g/L yeast extract, and the pH was adjusted to 7.0 ± 0.2 by adding HCl or NaOH. DOX was purchased from the Stanford Chemicals Company.

### Experimental Evolution of *Escherichia coli* Under DOX Stress

The evolution experiment consisted of two groups: a control group and a treatment group. In the control group, the medium did not contain DOX. The WT strain was grown in blank medium for 24 h and then subcultured in fresh medium with 1% inoculum. In the treatment group, the WT strain was grown in increasing concentrations of DOX for 38 successive subcultures. To start the experiment, the WT strain was grown for 24 h in medium containing 2 μg/ml DOX and then subcultured in another medium containing an increased DOX concentration with 1% inoculum. The DOX concentration was increased in increments of 2 μg/ml up to a final concentration of 76 μg/ml. Each subculture of the two groups were stored with 15% glycerol in a −80°C freezer for subsequent testing. The even-numbered successive subcultures were streaked onto LB plates for revival and then used to determine the MIC and IC_50_ (half maximal inhibitory concentration) of DOX. The 0th subculture (strain W0), 20th subculture (strain M20), and 38th subculture (strain E38) were chosen for streaking onto LB agar media plates. Three colonies were picked from the plates for morphological observation, biological function determination, genome sequencing, and transcriptome sequencing.

### MIC and IC_50_ Determination

According to standards of the Clinical and Laboratory Standards Institute (CLSI), the DOX standard was dissolved in sterile water to the standard solution (2,000 μg/ml). Then, the standard solution was diluted with LB broth into 19 mediums whose concentrations were 192, 128, 96, 64, 48, 32, 24, 16, 12, 8, 6, 4, 3, 2, 1.5, 1, 0.75, 0.5, and 0.375 μg/ml, respectively. The 19 DOX mediums were added to sterile 96-well polystyrene plates at 100 μl per well and were made into two assay plates. The DOX concentrations in wells 1–11 of the first assay plate were 192, 128, 96, 64, 48, 32, 24, 16, 8, 4, and 2 μg/ml, respectively. And the concentrations in 1–11 wells of the other assay plate were 12, 8, 6, 4, 3, 2, 1.5, 1, 0.75, 0.5, and 0.375 μg/ml, respectively. The 12th well of the two plates was used as a blank control (LB broth containing no antibiotics).

The fresh bacterial solution was diluted to an OD_600_ of 0.5 using 0.9% saline. The diluent was diluted five times with LB broth and added to each well of a 96-well plate. The 96-well plate was sealed and incubated at 37°C for 24 h on a shaker at 140 rpm. The OD_600_ value was measured with a microplate reader, the MIC value was recorded, and the IC_50_ was calculated using GraphPad Prism 7.0 software.

### Detection of the Biological Characteristics of *Escherichia coli*

Strains W0, M20, and E38 were selected, cultured to an OD6_00_ of 0.5, and inoculated in LB medium, at an inoculation amount of 1%. The cells were cultured in 96-well microplates. A total of 200 μl of culture medium per well was shaken and cultured at 37°C. The OD_600_ of the culture was measured by an enzyme-labeled instrument at 0, 4, 8, 12, 16, 20, 24, 28, 32, 36, 40, 44, 48, 52, and 56 h. The OD_600_ value indicates the cell density of the culture. The abscissa is the time, and the ordinate is the OD_600_ value, reflecting the difference in the growth characteristics of the strain. And the strains were applied to LB agar plate. After 24 h of culture (37°C), the colonies on the culture plate were photographed by an imager (Tanon 4600SF) to analyze the colony size. Crystal violet staining was used to detect the amount of *E. coli* biofilm formation. Nine replicates were examined for each strain. Data analysis was performed on SPSS 22 software (*N* = 81, one-way ANOVA, significance accepted at *p* < 0.05).

The BIOLOG microplate method was used to determine the intensity of carbon metabolism of *E. coli* (Khalil and Alsanius, 2009). The sample solution was cultured to an OD_600_ of 0.5 and diluted 1:1,000 with physiological saline to obtain a sample dilution. A total of 150 μl of the sample dilution was inoculated per well. The inoculated ECO plates were wrapped in tin foil (protected from light) and placed in a 25°C biological incubator. The absorbance values of the ECO plate at 590 (color and turbidity) and 750 nm (turbidity) were measured on an enzyme-labeled instrument at 0, 24, 48, 72, 96, 120, 144, and 168 h. The OD_590_ and OD_750_ values for each well were subtracted from the OD_590_ and OD_750_ values of the control well, respectively, and the actual OD_590–750_ values of each well were obtained (values less than 0.06 were treated as 0). OD590–750 values indicate the metabolic intensity for each carbon source. Average well color development (AWCD) indicates the average metabolic capacity for the total carbon sources. AWCD of each well = ∑ (OD_590–750_)/31 (31 carbon sources in the ECO plate). Single carbon source metabolism ratio = (single carbon source OD_590–750_) × 100%/∑ (OD_590–750_ of each carbon source). The total carbon sources included six major carbon sources: esters, amines, acids, carbohydrates, alcohols, and amino acids. Nine replicates were examined for each strain. Data analysis was performed on SPSS 22 software (*N* = 27, one-way ANOVA, significance accepted at *p* < 0.05).

### Whole-Genome Sequencing and Single-Nucleotide Polymorphism Analysis of *Escherichia coli*

The genome was sequenced with MPS (massively parallel sequencing) Illumina technology. A paired-end library with an insert size of 350 bp was constructed. The 350-bp library was sequenced using an Illumina HiSeq 4000 by the PE150 strategy. Library construction and sequencing was performed at Beijing Novogene Bioinformatics Technology Co., Ltd. Quality control of paired-end reads was performed using an in-house program. The original figure data obtained by Illumina HiSeq 4000 analysis were transformed into raw sequence reads (raw data or raw reads) by CASAVA base calling and stored in FASTQ (fq) format, containing sequencing information and the corresponding sequencing quality information of the reads. The sequence data were filtered, and the adapter and low-quality sequences were removed, resulting in clean data that were used for subsequent analysis.

Read comparison is the basis of resequencing analysis. Differences between the sample and reference can be identified by aligning the sample reads with the designated reference sequence. Mapping of reads to the reference sequence was performed using BWA software, and determination of the reference sequence coverage by the reads and analysis of the alignment results were performed using SAMtools software (Li et al., 2009).

Single-nucleotide polymorphisms (SNPs) are DNA sequence polymorphisms that are caused by single-nucleotide variations at the genome level, including those caused by transition, transversion, etc. InDel refers to the insertion and deletion of small fragments in the genome. SAMtools was used for detection of individual SNPs and InDels (<50 bp), as well as for analysis of the genome. SV (structural variation) refers to insertion, deletion, inversion and translocation of large segments at the genome level. Insertion (INS), deletion (DEL), inversion (INV), intrachromosomal translocation (ITX), and interchromosomal translocation (CTX) between the reference and the sample were detected by BreakDancer software (Chen et al., 2009).

The variation map of the whole genome was created by Circos to show read coverage and the distribution of SNPs and InDels.

Raw genome sequence data are available from NCBI (National Center for Biotechnology Information) (GenBank accession nos. SRR7100192, SRR7092030 and SRR7067320).

### Transcriptome Sequencing and Analysis of *Escherichia coli*

Total RNA of *Escherichia coli* was extracted using a E.Z.N.A Bacterial RNA Kit (R6950-01, OMEGA, United States) according to the manufacturer’s instruction. A total of 3 μg of RNA per sample was used as input material for RNA sample preparation. Sequencing libraries were generated using the NEBNext^®^ Ultra™ Directional RNA Library Prep Kit for Illumina^®^ (NEB, United States) according to the manufacturer’s recommendations, and index codes were added to attribute sequences to each sample. Ribo-Zero rRNA Removal Kit (Bacteria; Illumina, MRZB12424) was used for removal of rRNA from total RNA preparations. Fragmentation was carried out using divalent cations under elevated temperature in NEBNext First Strand Synthesis Reaction Buffer (5×). First strand cDNA was synthesized using random hexamer primers and M-MuLV reverse transcriptase (RNaseH-). Second-strand cDNA synthesis was subsequently performed using DNA polymerase I and RNase H. In the reaction buffer, dNTPs with dTTP were replaced by dUTP. Remaining overhangs were converted into blunt ends *via* exonuclease/polymerase activities. After adenylation of 3′ ends of DNA fragments, NEBNext adaptors with hairpin loop structures were ligated to prepare the samples for hybridization. To preferentially select cDNA fragments that were 150–200 bp in length, the library fragments were purified with the AMPure XP system (Beckman Coulter, Beverly, United States). Then, 3 μl of USER enzyme (NEB, United States) was used with size-selected, adaptor-ligated cDNA at 37◦C for 15min, followed by 5min at 95◦C before PCR. Then, PCR was performed with Phusion high-fidelity DNA polymerase, universal PCR primers, and Index (X) primer. Finally, products were purified (AMPure XP system), and library quality was assessed on am Agilent Bioanalyzer 2100 system.

Clustering of the index-coded samples was performed on a cBot cluster generation system using the TruSeq PE Cluster Kit v3-cBot-HS (Illumina) according to the manufacturer’s instructions. After cluster generation, the libraries were sequenced on an Illumina HiSeq platform, and paired-end reads were generated. Raw data (raw reads) in fq format were first processed with in-house Perl scripts. Simultaneously, Q20, Q30 and GC content were calculated, and clean data were obtained. All the downstream analyses were based on the high-quality clean data.

The reference genome and gene model annotation files were downloaded from the genome website directly. Both index construction of the reference genome and alignment of clean reads to the reference genome (*E. coli* K-12 BW25133, NZ_CP009273.1) were performed by using Bowtie2–2.2.3 (Langmead and Salzberg, 2012). HTSeq v0.6.1 was used to count the reads mapped to each gene. In addition, the FPKM of each gene was calculated based on the length of the gene and the number of reads mapped to the gene (Trapnell et al., 2009). Differential expression analysis of two conditions/groups (two biological replicates per condition) was performed using the DESeq R package (1.18.0) (Anders and Huber, 2010, 2013; Wang et al., 2010). The resulting *p* were adjusted using Benjamini and Hochberg’s approach for controlling the false discovery rate (Mao et al., 2005). Genes with adjusted *p* < 0.05 found by DESeq were designated as differentially expressed.

Gene Ontology (GO) enrichment analysis of differentially expressed genes was performed by the GOseq R package (Young et al., 2010). In addition, we used KOBAS software to test the statistical enrichment of differentially expressed genes in the KEGG pathways (Minoru et al., 2008).

## Results and Discussion

### Resistance Evolution and Morphological Changes in *Escherichia coli* Under DOX Stress

**Figure 1:**
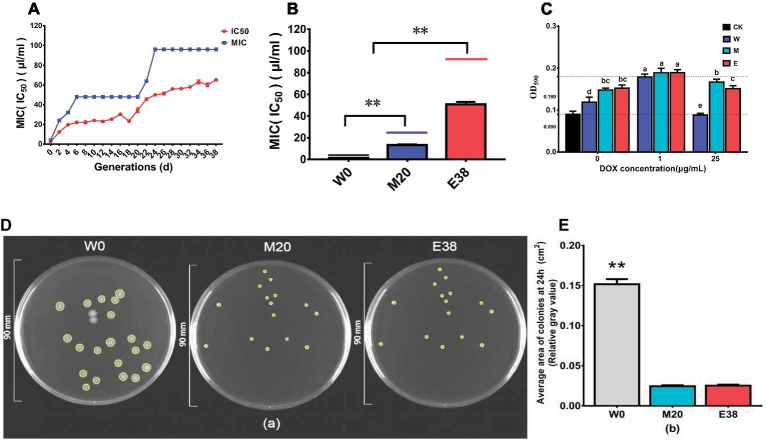
DOX resistance of different strains. **(A)** Trends of the MIC and IC_50_ values in the evolution experiment. **(B)** MIC and IC_50_ values of different strains. **(C)** The biofilm formation of the strains. **(D,E)** The colony area of the strains.

The overall MIC value of DOX with *E. coli* increased in the evolution experiment (Figure 1A). At the beginning of the experiment, the MIC value of DOX with *E. coli* was 4 μg/ml, and as the evolution experiment progressed, the MIC value increased significantly before the 6th day of acclimation. From the 6th day to the 20th day, the MIC value of the treatment group remained constant. However, after the 20th day, the MIC value rapidly increased again and reached 96 μg/ml on the 24th day, remaining stable at this value thereafter. The IC_50_ value of the control group remained basically constant, while the IC_50_ value of the treatment group exhibited a gradual increase (Figure 1A). The mean IC_50_ value of the treatment group increased from 2.18 ± 0.08 to 64.80 ± 0.75 μg/ml. The above results are consistent with the results of (Toprak et al., 2011), and it was found that DOX can induce an increase in resistance in *E. coli*. This result also needs to draw our attention. DOX is widely used in animal husbandry, and high concentrations of DOX can be detected in environmental media such as swine manure, soil and water bodies. We also need to pay attention to the effects of residual DOX on environmental microbial resistance.

To further observe changes in resistance, the 0th-passage sensitive strain W0, the 20th-passage moderately resistant strain M20 and the 38th-passage highly resistant strain E38 were selected for further analysis; the MICs of DOX with these strains were 4, 24, and 96 μg/ml, respectively, and the IC_50_ values of DOX were 1.31 ± 0.09, 13.17 ± 0.55, and 50.83 ± 2.36 μg/ml, respectively (Figure 1B). And the biofilm formation of strains M20 and E38 was significantly higher than that of the sensitive strain W0 (*p* < 0.05) (Figure 1C), indicating that the increase of biofilm formation was one of the pathways for *E. coli* to tolerate DOX. In addition, it was very interesting to find that the colony area of the resistant strains M20 and E38 was significantly smaller than that of sensitive strain W0 (*p* < 0.05) (Figures 1D,E). The size and motility of the colonies can reflect the metabolic capacity of the strain. Under the same conditions, the smaller the area of the strain is, the weaker its metabolic capacity (Shapiro, 1995). There may be a correlation between DOX resistance and metabolism capacity in *E. coli*.

### Changes in the Genomes of *Escherichia coli* Under DOX Stress

The genomes of the original susceptible strain W0 and resistant strains M20 and E38 were sequenced in this study. As a reference genome, we used the annotated supercontigs maintained by NCBI (NZ_CP009273.1). Contrary to our initial expectations, there were no SNPs or InDels in promoter regions, transcriptional regulators, or sensors. Compared with strain W0, there were only three mutant genes in the genome of strain M20 and six mutant genes in the genome of strain E38 (Table 1). Among them, the heme lyase gene (RS11460) is membrane proteins related gene. The 30S ribosomal protein S10 (RS17215), IS5 transposase (RS03405), and IS30 transposase gene (RS07365) are translation-related genes. The results are consistent with the result of Toprak (Toprak et al., 2011). *E. coli* and DOX were used as the research object and found that SNPs, which were mainly distributed on translation and membrane protein genes, played an important role in the evolution and prediction of microbial resistance (Toprak et al., 2011). The site of microbial transcription occurs at the ribosome. The ribosome is the target of DOX. DOX binds to the 30S ribosomal protein and affects protein synthesis, thereby inhibiting bacterial growth (Nonaka et al., 2005). Coincidentally, the mutant RS17215 encodes the 30S ribosomal protein. Mutations in the 30S ribosomal protein gene may result in a decrease in the affinity of DOX to the target site, thereby increasing the resistance of *E. coli* to DOX.

**Table 1:** Single nucleotide polymorphisms (SNPs) found in the resistant strains.

Both M20 and E38				
Gene ID	Annotation	Codon mutated	Amino acid position	Amino acid change
RS17215	30S ribosomal protein S10	GTC<->ATC	58	V<->I
**M20 only**				
RS17640	Sensory histidine kinase in two-component regulatory system	CCG<->CTG	248	P<->L
RS08025	Multiple antibiotic resistance transcriptional regulator	GGC<->GAC	55	G<->D
**E38 only**				
RS19705	UDP-N-acetyl-D-mannosaminuronic acid transferase	TTG<->GTG	134	L<->V
WGM003708	Hypothetical protein	AAT<->CAT	56	N<->H
		ATT<->ATG	59	I<->M
		ATT<->ATA	–	I<->I
RS03405	IS5 family transposase ISKpn26	TCT<->TCG	–	S<->S
RS11460	Heme lyase	CCC<->CCA	–	P<->P
RS07365	IS30 transposase	AAT<->AAC	–	N<->N

### Changes in the Transcriptional Profile of *Escherichia coli* Under DOX Stress

To further observe the changes in *E. coli* in the evolutionary experiment, we employed a comparative transcriptomic approach by sequencing ribosomal RNA-depleted total RNA from strains W0, M20 and E38 in the three media, i.e., the blank medium, 1 μg/ml DOX medium, and 25 μg/ml DOX medium. The 25 μg/ml DOX medium was used only for strains M20 and E38, since the strain W0 could not grow in this medium. A total of 4,774 genes was annotated, and the expression levels of these genes were determined (Sheet 1), including the four synonymous mutants of RS17215, RS17640, RS08025 and RS19705. According to the results, the expression of RS17215, RS17640 and RS08025 of strain W0 was significantly upregulated in the 1 μg/ml DOX medium. Similarly, the expression of the three genes of strains M20 and E38 was also significantly upregulated in the 25 μg/ml DOX medium. It can be inferred that mutation of RS17215, RS17640 and RS08025 in strains M20 and E38 plays an important role in the improvement of resistance.

**Figure 2:**
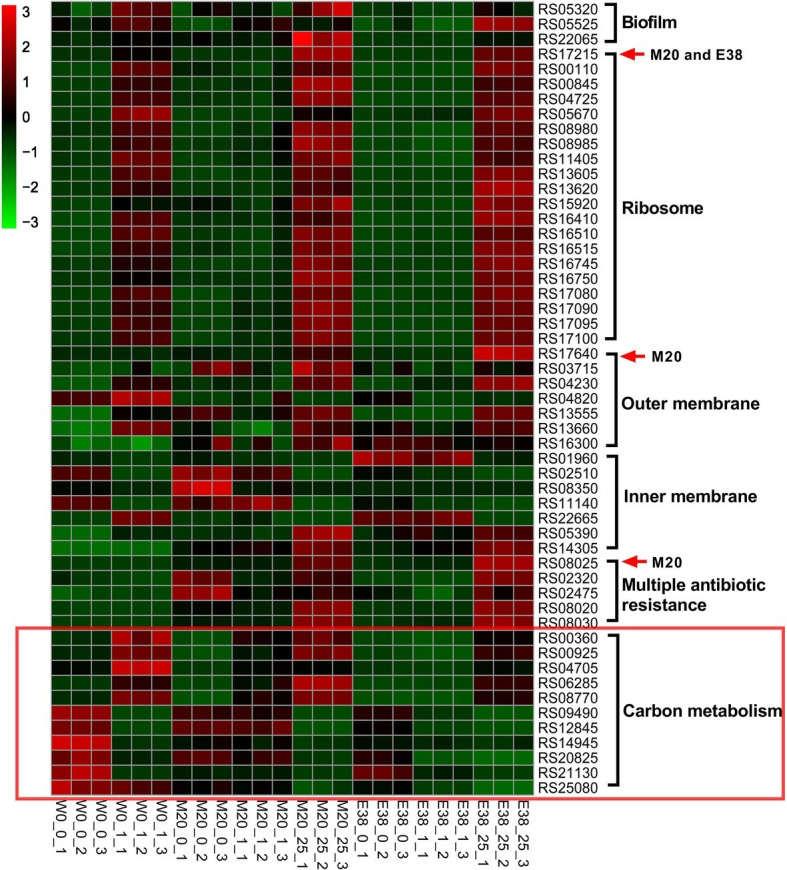
Transcriptomic and genomic information for genes involved in DOX resistance. The greener the square on the heat map is, the lower the expression level of the gene, and the redder the square is, the higher the expression level of the gene. The mutated genes in strain M20 or E38 are indicated by red arrows. The ordinate represents the gene ID. The abscissa indicates the grouping, wherein the second numbers, i.e., 0, 1, and 25, indicate the concentration of DOX (0, 1, and 25 μg/ml, respectively), and the third number indicates the number of repetitions.

Then, the differentially expressed genes were analyzed by GO and KEGG functional annotation. The mutant genes RS17215, RS17640, and RS08025 were found to participate in ribosomal biosynthesis, outer membrane biosynthesis, inner membrane biosynthesis and multiple-antibiotic resistance (Figure 2). Based on the results of biofilm formation, colony morphology and genome, the evolution of *E. coli* resistance to DOX was mainly through the increase of ribosome target mutations, and changes in cell membrane permeability (Nonaka et al., 2005; Patel, 2005; Ghai and Ghai, 2018). Among them, ribosome mutation is a classical tetracycline resistance pathway of bacteria (Nguyen et al., 2014), and reducing cell membrane permeability is a multi-drug resistant pathway (Lin et al., 2015). The changes in ribosomes and cell membranes could affect the protein synthesis and the entry of substances into the cells (MacDonald et al., 1967; Ekiert et al., 2017), which may further affect the carbon metabolism. In addition, the expression levels of carbon metabolism-related genes of resistant strains M20 and E38 were significantly lower than those of sensitive strain W0 (*p* < 0.05) (Figure 2). This result is consistent with the results of colony size, indicating that there was a relationship between DOX resistance and metabolism. However, we did not find mutations in related genes on the genomes. This result was consistent with our expectations. Under the stress of DOX, the mutations of *E. coli* were directionally selected and the mutations related to DOX resistance were retained (Kalia, 2015). This also suggested that these mutations may not be directly related to carbon metabolism.

**Figure 3:**
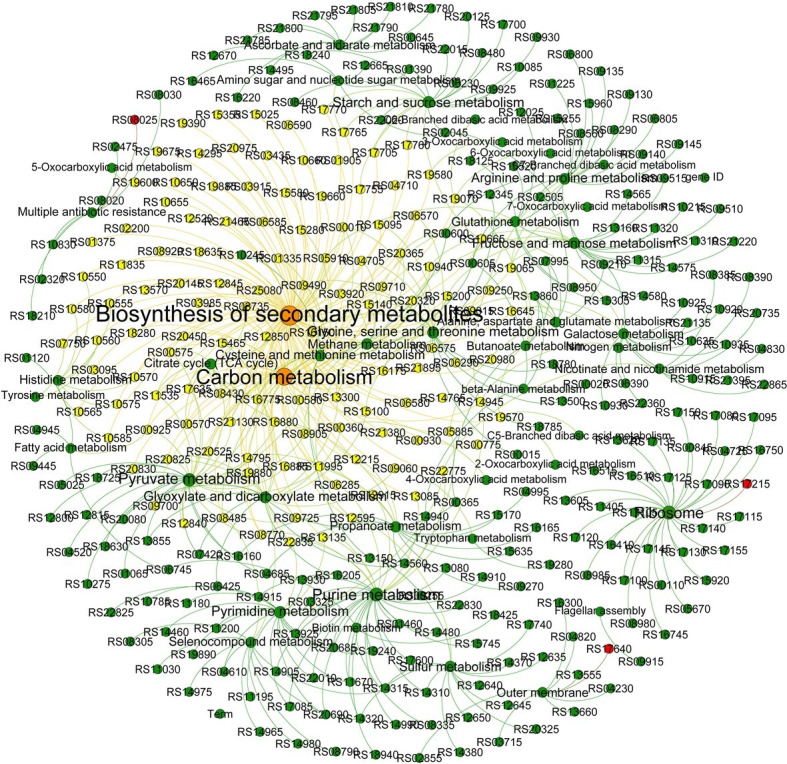
Network analysis revealing the co-occurrence patterns in the pathways. The node size represents the number of genes assessed, and the larger the node, the greater the number of genes. The red nodes indicate the mutant genes. The directional lines indicate the pathway in which the genes are involved. The genes associated with the carbon metabolism and biosynthesis of secondary metabolites are connected by the yellow directional lines.

Therefore, whether the mutated gene induced by DOX affects other non-mutant genes, and thus affects the differences in carbon metabolism-related pathways. Network analysis was used to analyze the genes with significant differences in expression (Figure 3). It was found that the genes associated with biosynthesis of secondary metabolites and carbon metabolism were also involved in histidine metabolism, Arginine and proline metabolism; pyruvate metabolism; galactose metabolism; methane metabolism; purine metabolism; oxocarboxylic acid metabolism; nicotinate and nicotinamide metabolism; selenocompound metabolism; alanine, aspartate and glutamate metabolism; fatty acid metabolism; glutathione metabolism; nitrogen metabolism; and glyoxylate and dicarboxylate metabolism. This result is consistent with the results of metabolic analysis, which showed that metabolism decreased significantly as resistance increased. But the mutant genes RS17215, RS17640 and RS08025 had no association with the genes involved in the above pathways, including biosynthesis of secondary metabolites and carbon metabolism. The relationship between microbial resistance evolution and carbon metabolism changes and its mechanism deserve further study.

### Changes in the Carbon Metabolism of *Escherichia coli* Under DOX Stress

**Figure 4:**
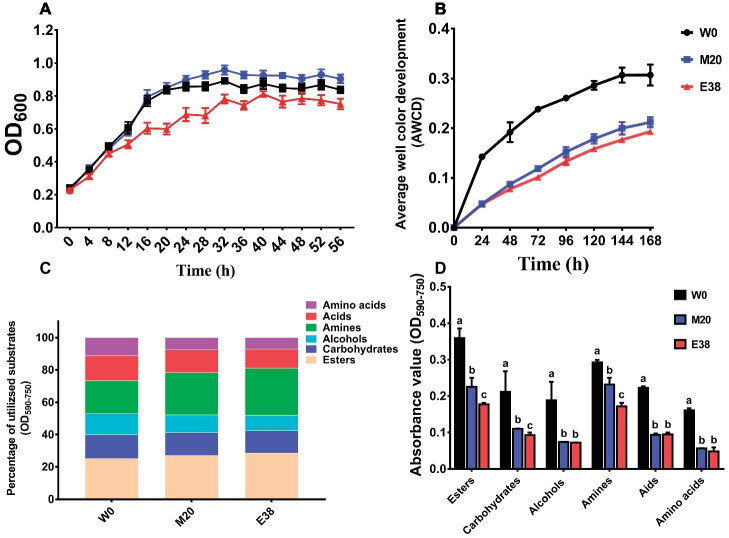
The average intensity of carbon metabolism in *E. coli*. **(A)** Growth density of strains in LB medium. **(B)** Average well color development (AWCD) was used as an indicator of changes in the average intensity of carbon metabolism of strains W0, M20, and E38. **(C)** Ratio of the metabolism intensity for different carbon sources. **(D)** AWCD at 72 h was used as an indicator of metabolic intensity for different carbon sources of strains W0, M20, and strain E38.

In the medium (LB) containing all the required carbon sources, the growth density of the resistant strains M20 and E38 was significantly lower than that of the sensitive strain W0 (Figure 4A), indicating that the metabolism of *E. coli* to total carbon sources decreases with the evolution of DOX resistance. Then, the BIOLOG microplate method was used to determine the intensity of carbon metabolism of *E. coli* (Khalil and Alsanius, 2009). AWCD was used as an indicator of changes in the average intensity of total carbon metabolism. The results showed that with culture growth, the AWCD of the three different strains exhibited a gradual increasing trend, increasing rapidly at the beginning and then gradually slowing down (Figure 4B). Moreover, strain W0 exhibited the highest metabolic intensity for six major carbon sources—esters, carbohydrates, alcohols, amines, acids and amino acids—followed by strains M20 and E38 (Figure 4D). However, the metabolic intensity ratio of different strains for the six major carbon sources was maintained in the same range, and the metabolic intensity for esters, amines, acids, carbohydrates, alcohols and amino acids accounted for 19.88–34.18%, 18.56–31.85%, 11.51–19.05%, 11.89–17.54%, 6.67–17.58%, and 2.26–14.78% of the total carbon sources, respectively (Figure 4C).

**Figure 5:**
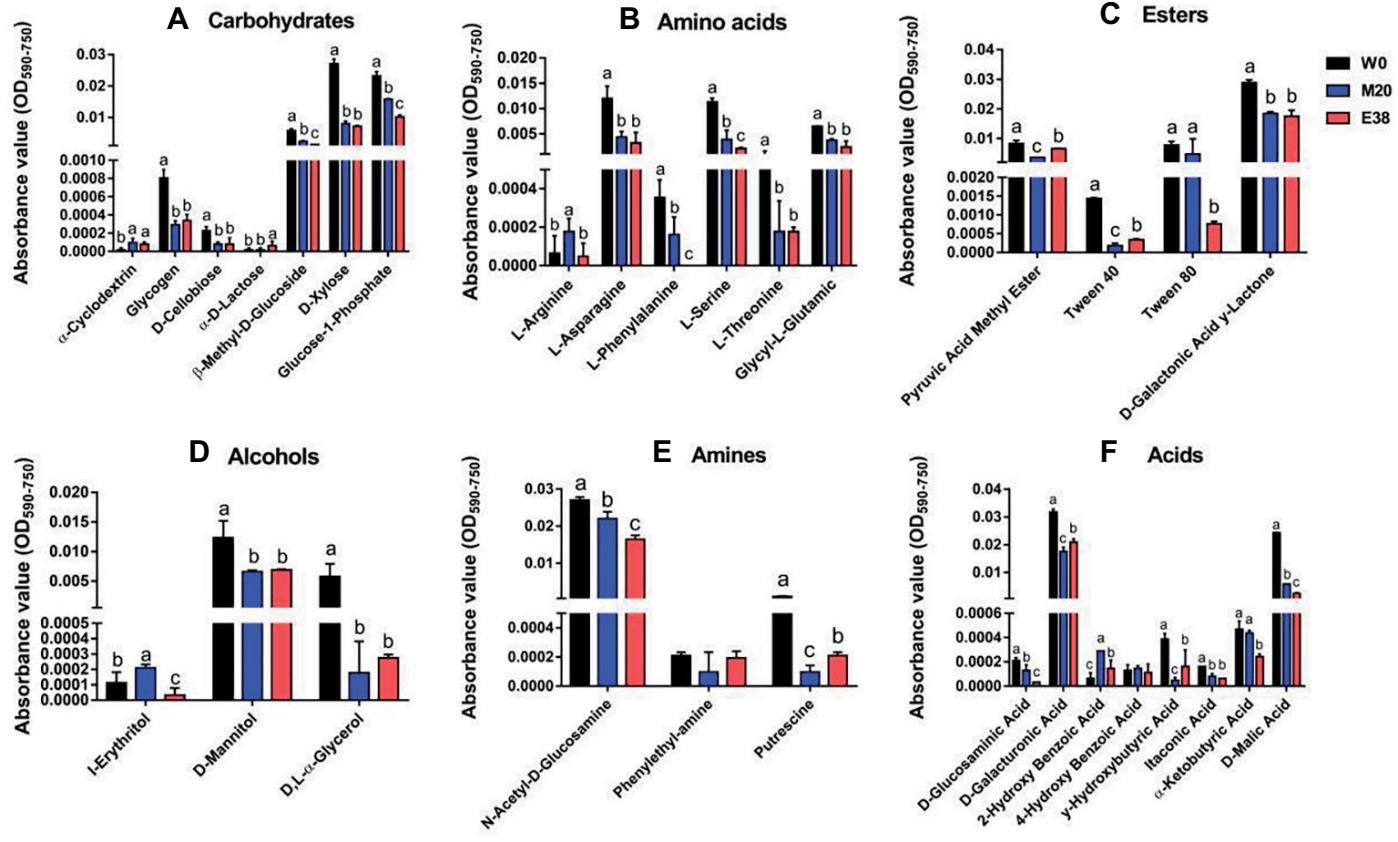
The metabolic intensity of 31 carbon sources in *E. coli*. **(A-F)** The metabolic intensity of carbohydrates, amines, acids, alcohols, esters and amino acids in *E. coli*.

The metabolism of *E. coli* to six types of 31 carbon sources was analyzed. It was found that, except for α-Cyclodextrin, α-D-Lactose, L-Arginine, I-Erythritol, Phenylethy-amine, 2-Hydroxy Benzonic Acid, 4-Hydroxy Benzonic Acid and α-Ketobutyric Acid, the metabolic intensity of strain M20 and E38 for the other 23 carbon sources were significantly lower than that of strain W0 (Figure 5). Studies have reported that the addition of glucose, mannitol, fructose and pyruvic acid and Fumaric acid can activate the relevant carbon metabolic pathway and increase the absorption of antibiotics into the cells (Barraud et al., 2013; Su et al., 2015; Meylan et al., 2017), increasing the sensitivity of bacteria (Allison et al., 2011). The results showed that enhancing the metabolism of related carbon sources was beneficial to the recovery of bacterial sensitivity. Therefore, the reduction of the relevant carbon metabolism level was conducive to the evolution of DOX resistance. However, its mechanism requires further research.

## Conclusion and Perspectives

This study demonstrated that under DOX stress, *E. coli* resistance increased, possibly due to ribosome target mutation, decreased cell membrane permeability, and increased expression of multiple efflux pumps. With the evolution of resistance, *E. coli* did not undergo carbon metabolism-related mutations, but the metabolism of 23 carbon sources was significantly reduced. The results presented here show that there may be a correlation between the evolution of *E. coli* DOX resistance and the decrease of carbon metabolism, but the mechanism was worthy of further research, providing a theoretical basis for the prevention and control of microbial resistance.

Considering high residuals of DOX in the environment, to comprehensively understand the evolution of microbial resistance and changes in metabolism in the environment, further detailed studies should be conducted in different environments. Of course, there are different types of antibiotics in clinical and production, and different antibiotics have different antibacterial mechanisms. Under the pressure of other antibiotics, whether microbes have the same resistance evolution and carbon metabolism changes require more comprehensive research.

## Data Availability Statement

The datasets generated for this study can be found in the NCBI (GenBank accession nos. SRR7100192, SRR7092030, and SRR7067320).

## Author Contributions

YWu and YY planned the project and designed the experiments. JM, JiL, XL, BM, YZ, YWa and JuL provided technical guidance for the test process. YY wrote the manuscript, which was critically reviewed by YWu and JM.

### Conflict of Interest

The authors declare that the research was conducted in the absence of any commercial or financial relationships that could be construed as a potential conflict of interest.
