# Corrigendum: Changes in the Carbon Metabolism of *Escherichia coli* During the Evolution of Doxycycline Resistance

**DOI:** 10.3389/fmicb.2022.852577

**Published:** 2022-02-08

**Authors:** Yiwen Yang, Jiandui Mi, Jiadi Liang, Xindi Liao, Baohua Ma, Yongde Zou, Yan Wang, Juanboo Liang, Yinbao Wu

**Affiliations:** ^1^College of Animal Science, National Engineering Research Center for Breeding Swine Industry, South China Agricultural University, Guangzhou, China; ^2^Ministry of Agriculture Key Laboratory of Tropical Agricultural Environment, South China Agricultural University, Guangzhou, China; ^3^Key Laboratory of Chicken Genetics, Breeding and Reproduction, Ministry of Agriculture, Guangzhou, China; ^4^Guangdong Provincial Key Laboratory of Agro-Animal Genomics and Molecular Breeding, South China Agriculture University, Guangzhou, China; ^5^Nanhai Office of Foshan Customs House, Foshan, China; ^6^Laboratory of Animal Production, Institute of Tropical Agriculture, Universiti Putra Malaysia, Serdang, Malaysia

**Keywords:** carbon metabolism, evolution, antibiotic resistance, DOX, *Escherichia coli*

In the original article, there was an error. The methods used to extract and purify RNA were not well described.

A correction has been made to **Materials and Methods**, **Transcriptome Sequencing and Analysis of** ***Escherichia coli*** section, paragraph 1:

“Total RNA of *Escherichia coli* was extracted using a E.Z.N.A Bacterial RNA Kit (R6950-01, OMEGA, USA) according to the manufacturer's instruction. A total of 3 μg of RNA per sample was used as input material for RNA sample preparation. Sequencing libraries were generated using the NEBNext^®^ Ultra™ Directional RNA Library Prep Kit for Illumina^®^ (NEB, USA) according to the manufacturer's recommendations, and index codes were added to attribute sequences to each sample. Ribo-Zero rRNA Removal Kit (Bacteria) (Illumina, MRZB12424) was used for removal of rRNA from total RNA preparations. Fragmentation was carried out using divalent cations under elevated temperature in NEBNext First Strand Synthesis Reaction Buffer (5×). First strand cDNA was synthesized using random hexamer primers and M-MuLV reverse transcriptase (RNaseH-). Second-strand cDNA synthesis was subsequently performed using DNA polymerase I and RNase H. In the reaction buffer, dNTPs with dTTP were replaced by dUTP. Remaining overhangs were converted into blunt ends via exonuclease/polymerase activities. After adenylation of 3′ ends of DNA fragments, NEBNext adaptors with hairpin loop structures were ligated to prepare the samples for hybridization. To preferentially select cDNA fragments that were 150–200 bp in length, the library fragments were purified with the AMPure XP system (Beckman Coulter, Beverly, USA). Then, 3 μl of USER enzyme (NEB, USA) was used with size-selected, adaptor-ligated cDNA at 37°C for 15 min, followed by 5 min at 95°C before PCR. Then, PCR was performed with Phusion high-fidelity DNA polymerase, universal PCR primers, and Index (X) primer. Finally, products were purified (AMPure XP system), and library quality was assessed on am Agilent Bioanalyzer 2100 system.”

The authors apologize for this error and state that this does not change the scientific conclusions of the article in any way. The original article has been updated.

## Publisher's Note

All claims expressed in this article are solely those of the authors and do not necessarily represent those of their affiliated organizations, or those of the publisher, the editors and the reviewers. Any product that may be evaluated in this article, or claim that may be made by its manufacturer, is not guaranteed or endorsed by the publisher.

